# A Study of the Effects of Moisture on Composite−to−Metal Double−Lap Shear Joints

**DOI:** 10.3390/ma17153841

**Published:** 2024-08-02

**Authors:** Weidong Li, Rui Zeng, Qian Zhang, Ziqi Duan, Pengfei Shen, Xiangyu Zhong, Shicai Jiang, Jianwen Bao

**Affiliations:** 1 National Key Laboratory of Advanced Composites, AVIC Composite Technology Center, AVIC Composite Corporation Ltd., Beijing 101300, China; liwdhappy@163.com (W.L.); dzq970920@163.com (Z.D.); 19821277882@163.com (P.S.); xyzhong2003@sohu.com (X.Z.); jiang_shicai@163.com (S.J.); 2 College of Architecture and Art, Hefei University of Technology, Hefei 230009, China; zq_hfut@hfut.edu.cn

**Keywords:** composite−to−metal joint, double−lap shear joint, wet effect, cohesive zone model, moisture stress

## Abstract

This work investigated the effects of moisture absorption treatment on composite−to−metal double−lap shear joints (DLSJs) bonded with epoxy adhesive film through experiments and simulations. The composite−to−metal DLSJ can be divided into five parts (the interface between the composite and adhesive, the interface between the adhesive and metal, the composite adherend, the metal adherend, and the adhesive layer). First, the wet−dependent properties of the adhesive and interfaces were obtained through adhesive tensile tests and GC tests, which showed that the properties of the adhesive and interfaces were significantly affected by the moist environment. Then, tensile tests of the composite−to−metal double−lap shear joints were carried out in dry and wet environments. Finally, based on the experimental investigations, a finite element (FE) model that considered cohesive damage was established for simulating damage evolution and predicting the failure loads and failure modes of the DLSJs. The results of both the experimental and numerical tests show that the DLSJ failure load decreases significantly after immersion in 95 °C water, and the major failure mode transfers from adhesive failure to interface failure. The research results provide a theoretical basis or basic data for the structural design of adhesively bonded composite−to−metal.

## 1. Introduction

Due to the widespread applications of carbon fiber reinforced polymer (CFRP) in various fields, connection structures consisting of CFRP and metal are rapidly developing in the aerospace, shipbuilding, and automotive industries [1,2,3,4,5]. The effective design and structural analysis of composite−to−metal joints are important in order to obtain good mechanical properties. Two types of composite−to−metal joints are mechanical connections (e.g., bolt connections) and adhesive connections. Compared with mechanical connections, the mechanical performance of adhesive connections is limited by the cohesive strength of the adhesive. However, adhesive connections can avoid galvanic corrosion and significant weight gain, making them the preferred choice for designers. In engineering applications, wet service environments are inevitable (e.g., aircraft flying in the rain and ships sailing on the sea). Therefore, guaranteeing the durability of composite−to−metal adhesively bonded structures in wet conditions has become a significant challenge for their applications in industrial fields [6].

The composite−to−metal adhesively bonded joint can be divided into five parts: the composite adherend, metal adherend, adhesive layer, composite/adhesive interface, and metal/adhesive interface. Many studies have focused on the mechanical properties of joints, the properties of adhesives, and bonding interface performance [7,8,9,10,11,12,13,14,15,16,17,18,19,20,21]. However, there are limited studies on the performance of composite−to−metal adhesively bonded joints in wet environments. Since adhesive is a main component of adhesive joints, the moisture absorption characteristics must be carefully considered when studying the effect of wet treatment on the joints. Liu et al. [6] designed dog bone specimens to study the influence of moisture on the tensile properties of epoxy adhesive. They found that the modulus of the epoxy adhesive decreased by approximately 23.6% and the tensile strength by 27% after immersion in water for 60 h. Loh et al. [11] investigated the mechanical performance of epoxy adhesive toughened with rubber after different moisture treatments. The results indicated that the mechanical performance, including the tensile modulus and strength, decreased by 38% and 53%, respectively. After moisture absorption, the hydrogen bonds between the adhesive molecular chains were destroyed, leading to the plasticization of the adhesive.

**Figure 1:**
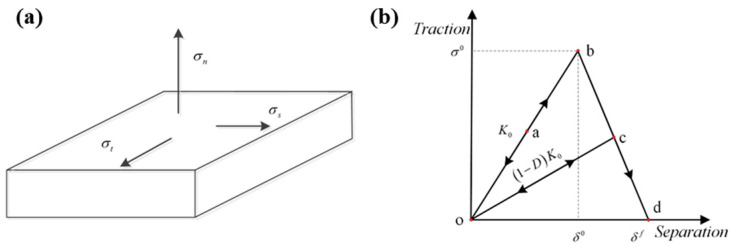
Schematic diagram of CZMs: (**a**) cohesive element, (**b**) bilinear traction–separation model.

To precisely investigate the interfacial behavior of composite/adhesive and metal/adhesive bonds, cohesive zone models (CZMs) were used to simulate the failure mode of adhesive joints [6,12,13,14,15,16,17]. Figure 1a shows how cohesive elements (CEs) under stress, split into shear (*σ_s_*), tangential (*σ_t_*), and normal (*σ_n_*) components, were utilized to analyze the CZMs. To depict the failure modes of CEs, a bilinear traction law (Figure 1b) was considered a simple method [15,16,17,18,19,20,21], which consisted of an elastic part with a softening stage. As shown in Figure 1b, the stiffness and strength of the cohesive interface are denoted as *K*_0_ and *σ*^0^, respectively, while the displacements at damage initiation and failure are denoted as *δ^0^* and *δ^f^*. The interlaminar fracture toughness (*G_C_*) can be represented by the area of the triangle marked *obd. G_C_* can be obtained from end notched flexure (ENF) and double cantilever beam (DCB) tests [18]. Shindo et al. [17] investigated the Mode I (crack opening mode) stratification of cloth/epoxy using DCB testing at −269.15 °C, −196.15 °C, and room temperature environments via the FEM and experimental schemes. The results show that the value of *G_C_* investigated for Mode I (*G_IC_*) decreases with a reduction in the test temperature. Balzani [18] obtained the fracture energy release rate of different modes through mixed−mode bending (MMB), DCB, and ENF tests. However, the abovementioned studies only simulated and analyzed the behavior of adhesive joints in dry environments.

Some investigations concentrated on the mechanical performance of composite−to−metal joints. However, the vast majority did not pay attention to the environmental influences on the properties of the joints [22,23,24,25,26,27]. Anyfantis [22,23,24] first investigated the influence of process conditions on the mechanical performance of single−lap joints through experiments. Then, a finite element method with CEs was conducted to simulate the failure process of the adhesive zone. The impact of the overlap length was the most significant on tensile stiffness and strength. Kum [25] studied the mechanical behavior of composite−to−metal double−lap shear joints (DLSJs) through experiments and numerical simulations, and the results showed that joint failure occurred in the first layer of laminates close to the adhesive regions.

To date, several studies have been carried out regarding the environmental effects on the mechanical properties of DLSJs. Knighta [28] investigated the impact of environmental aging on the mechanical properties of single−lap shear specimens (SLSSs). The results showed that the strength significantly decreased after environmental treatments, with the failure mode evolving to fiber tearing from cohesive tearing. In their study, Liu et al. [6] reported that the composite double−lap shear joints lost about 31.3% of the joint strength when immersed in hot water (90 °C) for 2.5 d, with multi−failure modes transferring to cohesive failure.

To the authors’ best knowledge, few results regarding the mechanical behavior of composite−to−metal adhesive joints after treatment under moist environments have been obtained so far. Moreover, the effects of moisture on the interlaminar properties of adhesively bonded structures have barely been reported, especially for composite/metal interfaces. This work investigated the influence of moisture on the mechanical properties of composite−to−metal DLSJs and their failure mechanisms under different hydrothermal treatments. First, a sequence of experiments involving adhesive tensile tests and *G_C_* tests were conducted to examine the mechanical properties of the composite/adhesive interface, the metal/adhesive interface, and the adhesive alone, both in wet and dry environments. Then, tensile experiments were conducted to obtain the strength and failure modes of the DLSJs in wet and dry environments. Based on these experiments, a progressive damage FE model was built to simulate the failure loads and modes of DLSJs under different environments, which incorporates the wet−dependent properties of adhesives and metal/composite interfaces.

## 2. Materials and Experiments

### 2.1. Materials and Specimens

The adhesive with epoxy as the main component used in this work was SY−14M−III, purchased from the Beijing Institute of Aeronautical Material. The basic physical properties are listed in Table 1.

**Figure 2:**
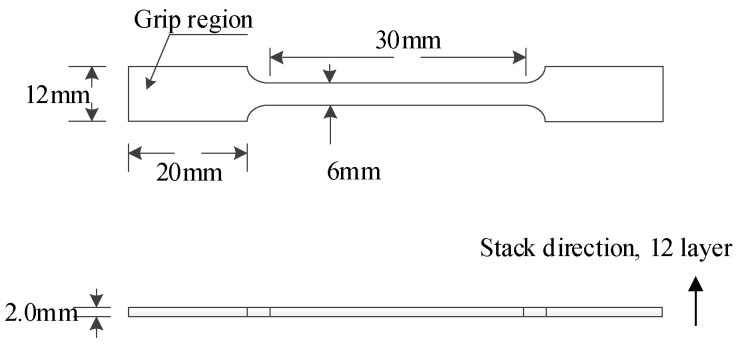
Geometry and dimensions of adhesive tensile specimens.

To examine the mechanical properties of the adhesive, 12 sheets of SY−14M−III film with a size of 300 mm × 300 mm were selected and spread into a piece of laminate. During the spreading process, each layer of film was ironed with an 80 °C electric iron to ensure a tight fit between the two layers. As far as possible, remove the large bubbles between the adhesive layers during this process. Note that immediate contact between the body and the film’s surface should be avoided throughout the spreading process. Finally, 12 layers of SY−14M−III were stacked together and cured to manufacture the adhesive laminates. Then, the laminates were cut into the shape shown in Figure 2.

**Figure 3:**
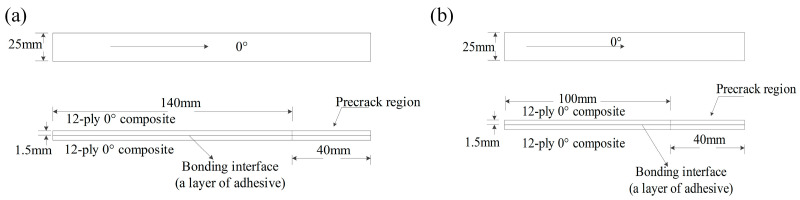
Geometry and dimensions of the composite/adhesive specimens: (**a**) DCB specimen, (**b**) ENF specimen.

To obtain the interface performance (the interlaminar fracture toughness) of the laminate (CCF800H/AC531, which were supplied by AECC BIAM, Beijing, China)/adhesive (SY−14M−III) specimens, the critical fracture energies G_IC_ and G_IIC_ were obtained by DCB and ENF tests according to ASTM D5528 [29] (*G_IC_*) and ASTM D7905 [30] (*G_IIC_*, i.e., crack sliding mode interlaminar fracture toughness), respectively. Figure 3 shows the geometric configurations of the laminate/adhesive specimens. An artificial delamination with a length of 40 mm was introduced using 0.01 mm of PTFE film [6,31].

**Figure 4:**
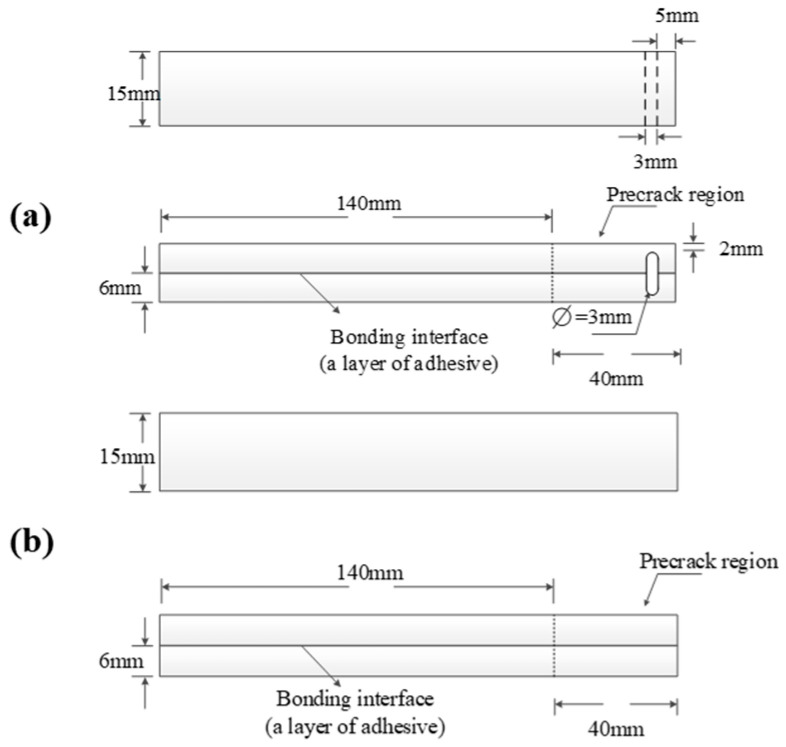
Geometry and dimensions of the metal/adhesive specimens: (**a**) DCB specimen, (**b**) ENF specimen.

Because there is no corresponding test standard (interlaminar fracture toughness) for metal/adhesive specimens, DCB and ENF specimens were manufactured, and corresponding tests were conducted as per ASTM D5528 and ASTM D7905. Figure 4 shows the geometric configurations of the metal/adhesive specimens made of two 6 mm thick metal plates bonded with adhesive [6,31]. The materials of the metal and adhesive were Al−7075−T6, anodized with phosphoric acid, and SY−14M−III. The metal was treated before the specimen was made to prevent the metal/adhesive specimen with fracture toughness from chemical reaction during moisture adsorption and to improve the bonding interface quality of the metal/adhesive specimens. Thus, the bonding strength between the metal and the adhesive will increase. If the earpieces used in the laminate/adhesive specimen were used in a DCB specimen of the metal/adhesive again, the earpieces would fall off first, while the interface of the metal/adhesive would remain undamaged. Therefore, we adopted a new method, making a small slot in the metal layer to insert a stronger metal bar. The thickness of the metal was also increased. The interlaminar damage of the metal/adhesive was induced by stretching the metal bar. Because only the adhesive layer in the specimen can absorb moisture, the width of the test specimens was designed to be 15 mm in order to reduce the moisture absorption time. The artificial delamination was also made using 0.01 mm of PTFE film.

**Figure 5:**
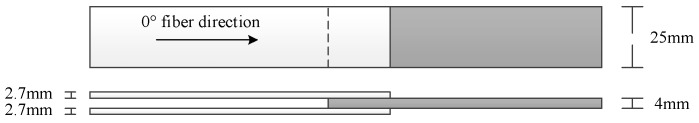
Geometry and dimensions of composite−to−metal DLSJ.

The metal/composite DLSJ specimens were manufactured, as shown in Figure 5, based on ASTM D3528 [32]. The coupon’s adherends were machined from 4 mm 136 Al−7075−T6 plates and 2.7 mm laminates ([45/0_2_/−45/90/45/0_2_/−45/0]_s_). The overlap length and overlap width are both 25 mm.

### 2.2. Experiments

#### 2.2.1. Moisture Absorption Tests

To study the impact of moisture on the adhesive and interfaces (*G_C_* for the laminate/adhesive interface and the metal/adhesive interface), tensile specimens of the adhesive and *G_C_* specimens of the metal/adhesive and laminate/adhesive specimens were bathed in deionized water at 95 °C for 140 h, 29 d, and 40 d, respectively, which in order to reach saturation moisture absorption, as wet group specimens. The composite−to−metal DLSJ specimens were soaked in 95 °C water for 50 d.

#### 2.2.2. Mechanical Tests

**Figure 6:**
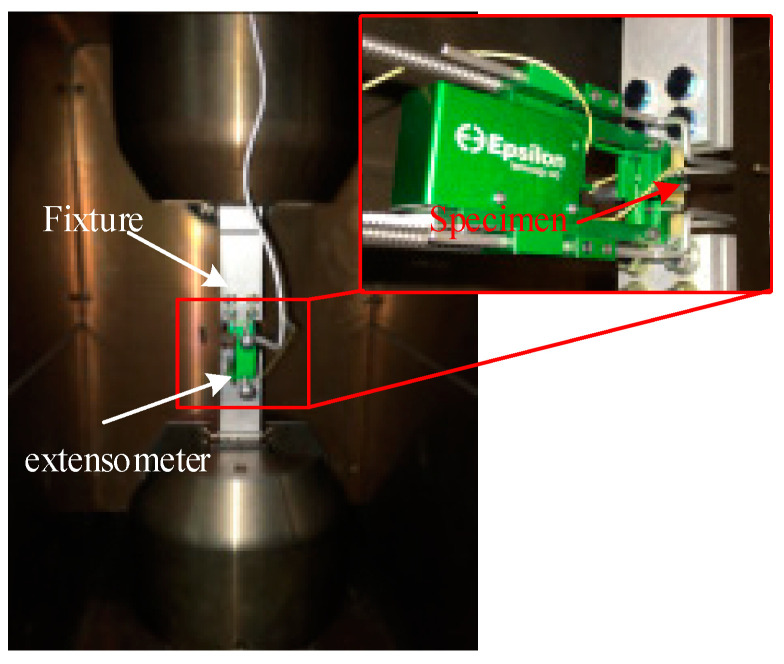
Tensile test for the adhesive.

A test machine, Instron 8801 (Instron Corp., Boston, MA, USA, precision 0.1%, and range 100 kN) equipped with an extensometer (precision 0.1%, span 40.8 mm) was used to test the tensile properties of the SY−14M−III adhesive at a 0.5 mm/min loading speed (Figure 6). The specimens were divided into two groups (wet and dry), each containing three specimens.

**Figure 7:**
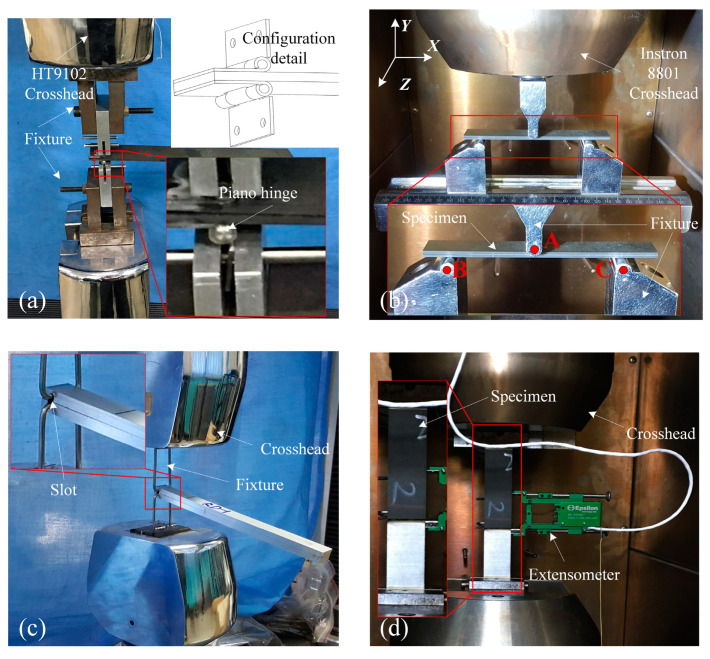
Test set-up for (**a**) DCB of composite/adhesive specimens; (**b**) ENF of composite/adhesive specimens; (**c**) DCB of metal/adhesive specimens; (**d**) tensile test of DLSJs.

For the *G_C_* specimens of the composite/adhesive bonded joints, HT9102 (HungTa Corp., Taipei, Taiwan, China, accuracy 0.1%, and range 100 kN) and Instron 8801 tools were used to conduct *G_IC_* tests (DCB) and *G_IIC_* tests (ENF), respectively. For the *G_IC_* tests (Figure 7a), vertical tension was applied to open the delamination of the specimen through the hinges on the end of the samples. In accordance with ASTM D5528, the loading speed was set at 3 mm/min. In the *G_IC_* test for the wet group, the hinges were bonded to the specimen after immersion in the water. For the *G_IIC_* tests, the three−point bending test was chosen, and the crosshead rate was maintained at 1.25 mm/min during the test, as shown in Figure 7b. During the test, the span (the displacement between point A and point B) was set to 101.6 mm, and the initial crack length (the displacement between point A and the crack tip along the X direction) was set to 25.4 mm. For the *G_C_* specimens of the metal/adhesive bonded joints, *G_IC_* and *G_IIC_* tests were also carried out using HungTa HT9102 and Instron 8801 equipment, respectively. For the *G_IC_* tests, the opening load was implemented on the DCB specimen using a special fixture with a 3 mm/min speed, as shown in Figure 7c. The set−up of the *G_IIC_* tests was the same as that for the composite/adhesive test, and the crosshead rate was also maintained at 1.25 mm/min throughout. The span and initial crack length were also set to 101.6 mm and 25.4 mm, respectively.

For the composite/metal DLSJ specimens (Figure 7d), the tests were conducted on an Instron 8801 equipped with an extensometer for measuring deformation. In accordance with ASTM D3528, the loading speed was set at 1.25 mm/min.

## 3. Results

### 3.1. Adhesive Tensile Tests

**Figure 8:**
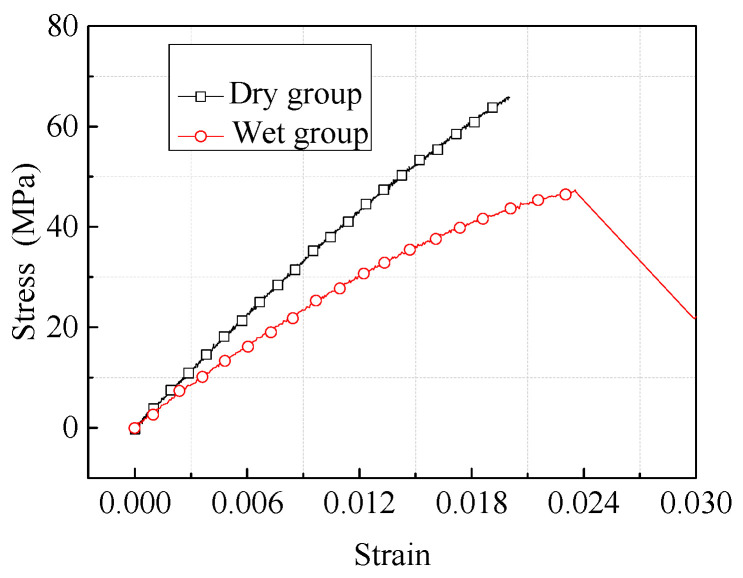
Stress−strain curves of adhesive under different environments.

The results of the adhesive tensile tests were concentrated on the tensile strength and modulus. The stress−strain curves of the adhesive specimens in dry and wet environments are compared in Figure 8. The wet environment has a significant negative impact on tensile strength and modulus, which decreased by approximately 28.1% (dispersion 3.8%) and 30.9% (dispersion 4.5%), respectively.

### 3.2. G_C_ Tests

**Figure 9:**
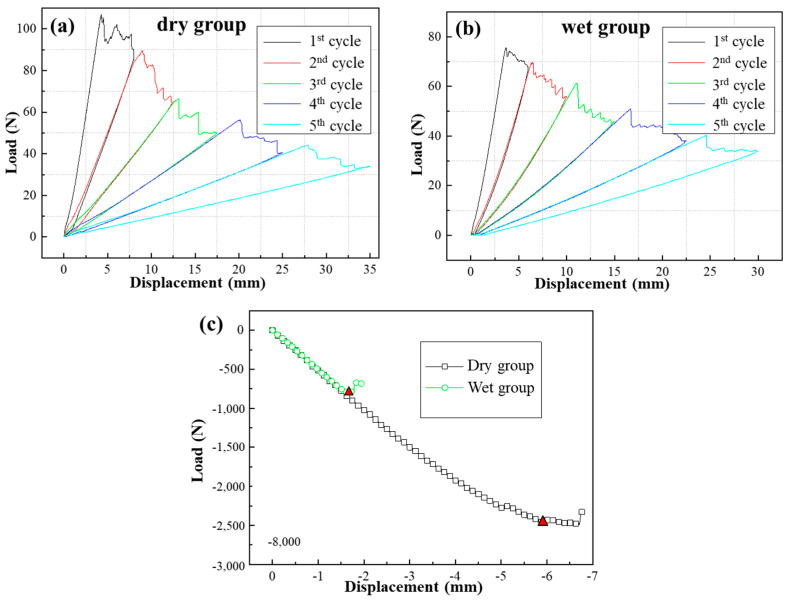
Load−displacement curves of composite/adhesive *G*_C_ tests: (**a**) DCB test result for dry group, (**b**) DCB test result for wet group, (**c**) ENF test result.

The load−displacement curves of the *G*_C_ tests under different environments are illustrated in Figure 9, and each curve of the *G*_IC_ specimens contains five cycles. Figure 9 shows that the ultimate loads of the composite/adhesive *G*_C_ tests significantly decrease after immersion in water. Table 2 summarizes the average values for the *G*_C_ specimens, which were calculated using the method shown in ASTM D5528 and ASTM D7905. It should be noted that the red triangles shown in Figure 9c were taken as the failure points for the ENF test. The *G*_IC_ of the wet group specimens decreased by approximately 32.6%, and the *G*_IIC_ reduced by 91.2%.

**Figure 10:**
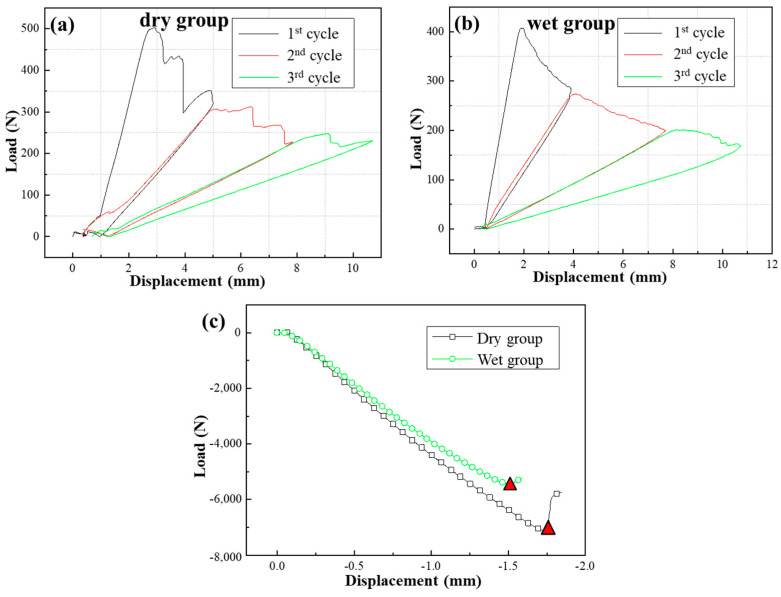
Load−displacement curves of metal/adhesive *G_C_* tests: (**a**) DCB test result for dry group, (**b**) DCB test result for wet group, (**c**) ENF test result.

Figure 10 illustrates the load−displacement curves of the metal/adhesive G_C_ tests under two different environments (dry and wet). Since the specimens with the metal/adhesive were completely delaminated and damaged after the third cycle, each curve of the *G_IC_* specimens contains three cycles. Figure 10 demonstrates that the ultimate loads of the metal/adhesive *G_C_* tests also exhibited significant degradation after immersion in water. The computing method used for the *G_C_* tests was the same as for the composite/adhesive interface *G*_C_ tests. As shown in Table 3, the *G_IC_* of the wet group specimens decreased by 33.5%, and the *G*_IIC_ reduced by 67.4%.

### 3.3. Double−Lap Shear Joints

**Figure 11:**
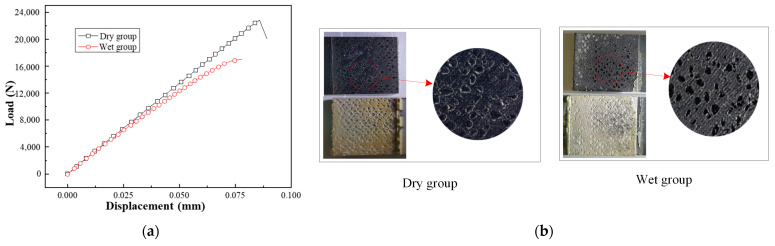
Experimental results of (**a**) load−displacement curves and (**b**) failure modes.

The load−displacement curves of the DLSJ samples under dry and wet environments are exhibited in Figure 11a, where the displacement was measured using a 40.8 mm span extensometer. The ultimate load of the wet group specimens decreased by approximately 25.4%, while the initial stiffness (the slope of the load−displacement curve) of the DLSJ specimens in different environments was nearly invariable, indicating that the moisture treatment significantly affects DLSJ strength and slightly influences stiffness. The failure modes corresponding to the different environments are different. Figure 11b shows the typical failure modes of the specimens under dry and wet environments. The main damage is adhesive failure in the dry group, while the main damage is interlaminar failure in the wet group. The same phenomenon can be noticed in the previous work [31].

## 4. FE Analysis

### 4.1. FE Model

**Figure 12:**
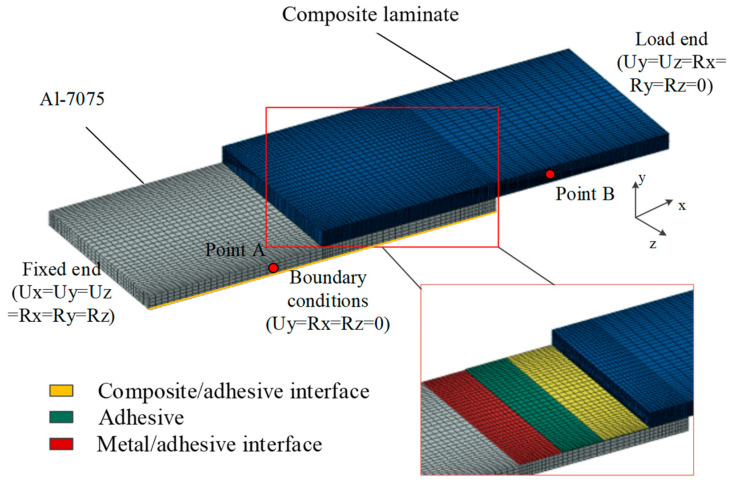
FE model and the details of DLSJ.

Figure 5 shows the geometry of the composite−to−metal DLSJ; thus, the details are not described here. Figure 12 shows the loading conditions, boundary conditions, and meshes of the FE model established in this work for the composite−to−metal DLSJ, using C3D8R (8−node linear solid element) in Abaqus/Standard. Here, 0−thickness conode cohesive elements were introduced to simulate the interfaces in the joint. Each layer of the laminates and adhesive was set as an independent unit, and the adhesive region of the DLSJ was cut into more fine meshes (minimum element size was 0.1 mm, and the number of elements was two in each layer). The finite model held 62,510 solid elements and 10430 cohesive elements in total. As the geometric and boundary conditions of the structure were symmetrical, half of the model along the thickness direction was established here. As shown in Figure 12, the outer side of the metal part was fixed, and the force along the x direction was applied to the end of the composite plate. Since the damage to the DLSJ and the effect of hygrothermal behavior occurred in the bonding region, the clamping region was far enough away from the bonding region, so the working section is taken to be 75 mm for numerical simulation. The distance between point A and point B was set as 40.8 mm, which corresponded to the span of the extensometer. The laminate parameters are shown in Table 4. Because the damage to the joint only occurred at the adhesive and interfaces in the experimental process, the degradations in the mechanical properties of the laminates were disregarded during the subsequent simulation. The Poisson’s ratio of Al−7075−T6 was 0.33, and the elastic modulus was 73,000 MPa. The stress−strain relationships of the adhesive were obtained via experiments, as shown in Figure 8.

### 4.2. Damage Criteria and Degradation Rules

**Figure 13:**
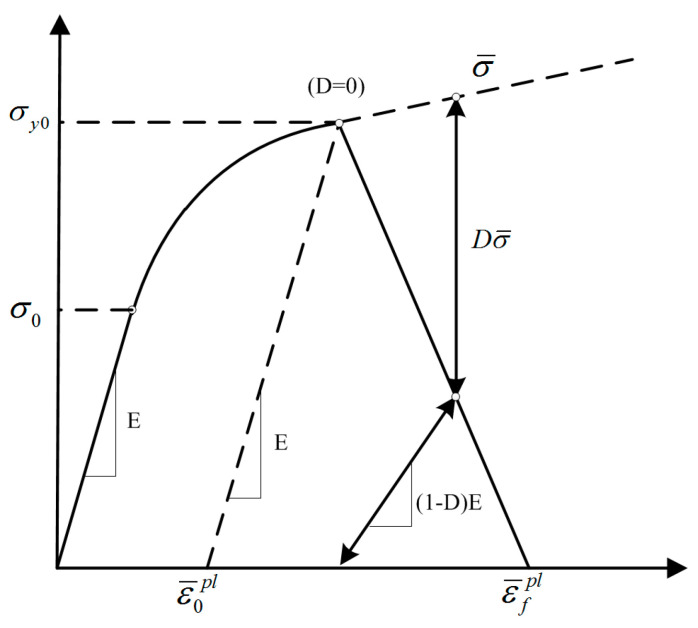
Mechanical behavior of adhesive with progressive damage degradation.

Figure 13 shows the mechanical behavior of the adhesive with gradual damage degradation. *σ_y_*_0_ is the maximum equivalent Mises stress, measured via the bulk adhesive test; *σ*_0_ is the yield strength of the adhesive; ${\bar{\varepsilon }}_{0}^{pl}$ is the plastic strain; and ${\bar{\varepsilon }}_{f}^{pl}$ is the elongation at break [33,34,35,36,37].

**Figure 14:**
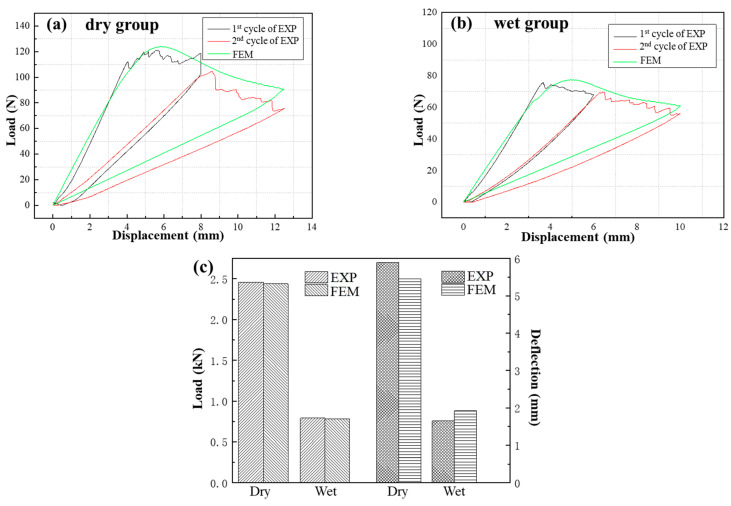
Comparison of EXP results and FE results for the composite/adhesive specimens: (**a**) DCB test results for the dry group, (**b**) DCB test results for the wet group, (**c**) ENF test results.

**Figure 15:**
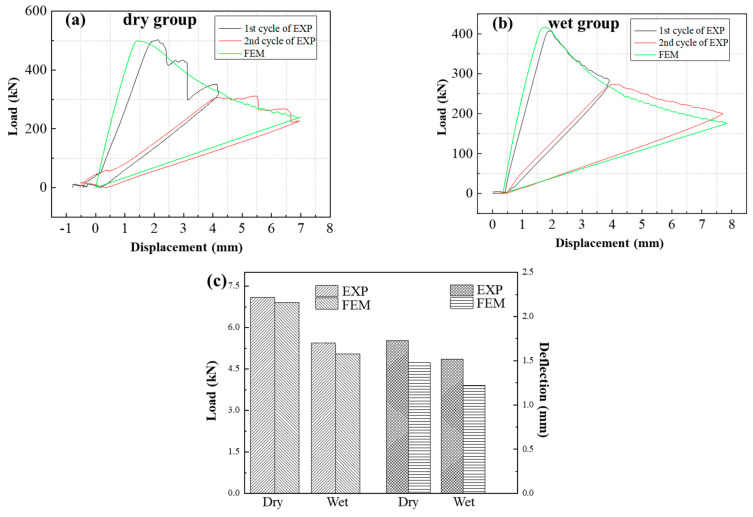
The comparison of EXP results and FE results of the metal/adhesive specimens: (**a**) DCB test results of the dry group, (**b**) DCB test results of the wet group, (**c**) ENF test results.

The following formula expresses the damage criterion and evolution law of CEs.

(1) $${\left\{\frac{\sigma_{1}}{\sigma_{1}^{0}}\right\}}^{2}+{\left\{\frac{\sigma_{2}}{\sigma_{2}^{0}}\right\}}^{2}+{\left\{\frac{\sigma_{3}}{\sigma_{3}^{0}}\right\}}^{2}=1\;G^{C}=G_{n}^{C}+\left(G_{s}^{C}-G_{n}^{C}\right){\left\{\frac{G_{s}+G_{t}}{G_{n}+G_{s}+G_{t}}\right\}}^{\eta }$$

where *G_n_*, *G_s_*, and *G_t_* denote fracture energy release rate components (*n*, *s*, and *r* refer to normal, shear, and tangential direction); *σ*_1_, *σ*_2_, and *σ*_3_ denote the stress components of CEs; $G_{n}^{C}$, $G_{t}^{C}$, and $G_{s}^{C}$ denote the critical *G_IC_* and *G_IIC_*, and *G_III__C_* (crack scissoring mode, which has similar mechanical properties with Mode II, i.e., *G_s_* = *G_t_* and *G_IIC_* = *G_IIIC_*) obtained from the tests above; and $\sigma_{1}^{0}$, $\sigma_{2}^{0}$, and $\sigma_{3}^{0}$ represent normal interface strength and interface shear strength in two directions, which cannot be obtained through tests. Thus, FE models introducing the cohesive zone model of DCB and ENF were established, and the properties of the composites used in the model were the same as those for the DLSJ [31]. The parameters of interface stiffness and interface strength in the laminate/adhesive and metal/adhesive specimens were inversed based on DCB and ENF tests. Table 2 and Table 3 show the parameters of interface stiffness and interface strength in the DCB and ENF model. Figure 14 and Figure 15 show the FE results for the composite/adhesive specimens, which have good consistency with the experimental results (EXP). During the DCB and ENF tests, a white fluorescent agent was applied to the side of the specimens, which attached a graduated soft ruler. Thus, the crack length can be read by photographing the sample with a high−speed camera. Because the crack lengths of both sides were asymmetric after the first loading−unloading cycle in the *G_IC_* test, two loading−unloading cycles were chosen to verify the FEM. A comparison of the crack evolution between the EXP and FEM results is shown in Table 5. It can be seen that the errors in the crack evolution between the test data and the FE prediction are within 13.7%. This indicates that the cohesive parameters can be used in later investigations to simulate the damage evolution of the interfaces.

### 4.3. The Stress−Strain Relationship under the Wet Environment

In Abaqus2021 [38], the calculation of wet stress cannot be solved directly. Therefore, in this paper, the following method was used to realize the hygro−mechanical coupling calculation in the finite element software Abaqus:

Due to the consistency of the governing equations of internal stresses caused by hygrothermal expansion, the thermal stress solution module to calculate the wet stresses was adopted in this paper. Firstly, on the basis of the moisture absorption model to get the wet concentration field, user−defined field and user−defined field variable subroutine (subroutine USDFLD) were used to import the wet concentration field into the result file (field variable.fil). Then, the field variables in the result file were rewritten to the temperature field result file. Next, the field variables in the result file were rewritten into the temperature field result file (temperature.fil). Finally, the obtained temperature field result file was introduced into the DLSJ model as the temperature load to calculate the internal stress caused by moisture.

Because the composite−to−metal DLSJ specimens were bathed in 95 °C deionized water for 55 d, it is assumed that both the laminate and adhesive reached saturation moisture absorption. The wet concentration field was applied to the structure as a predefined field. The strain introduced by moisture expansion can be expressed as follows:

(2) $$\left\{\varepsilon \right\}=\left[C\right]\left\{\sigma \right\}+\left\{\alpha \right\}\Delta c$$

The following formula shows the constitutive equation of wet elasticity.

(3) $$\left\{\sigma \right\}=\left[D\right]\left\{\varepsilon \right\}-\left[D\right]\left\{\alpha \right\}\Delta c$$

where [*C*] denotes the compliance matrix, [*D*] denotes the elastic matrix, {*α*} denotes the moisture expansion coefficient vector, and *Δc* denotes the increase in wet concentration (%).

Research [36] has found that the expansion coefficient of epoxy adhesive is 0.0016–0.01 per 1wt% moisture absorption. According to the present paper, the expansion coefficient was tested as 0.0022 per 1wt% moisture absorption [6,31]. Based on references [6,39,40], the longitudinal and transverse expansion coefficients of the composites (*α*_11_ and *α*_22_) were set as 2.5 × 10^−8^/wt% and 0.001/wt%, respectively. The saturated moisture content values for the adhesive and composite were 0.077 and 0.012, respectively [6].

**Figure 16:**
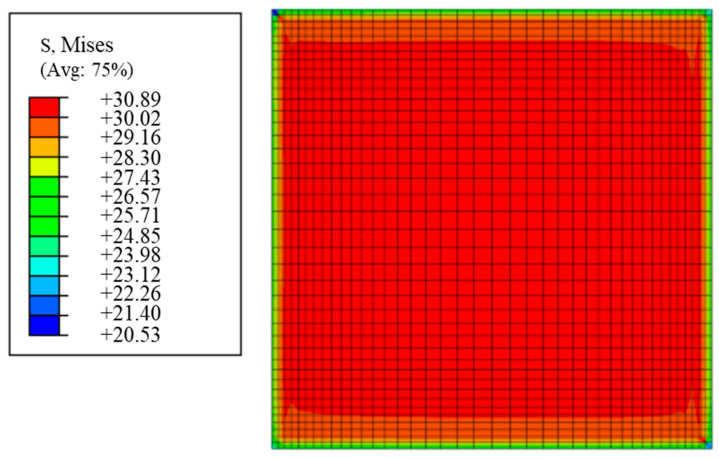
The Mises stress distribution in the adhesive.

Figure 16 shows the Mises stress distribution of the adhesive under a wet environment, from which the maximum stress appears at the inner part of the adhesive, and the values are less than the critical stress. This suggests that the cracks observed in the DLSJ tests were generated during the tensile process and not during the hygroscopic process. It can also be known that the stress distribution of the adhesive after moisture absorption was not uniform, and it is necessary to add pre−stress to the adhesive in the DLSJ model.

### 4.4. FE Results for DLSJs

Because the DJSL specimens were manufactured by artificial bonding, the bonding length of the DJSLs cannot be controlled. Therefore, the ultimate load of the DJSLs was not a reference value when verifying the finiteness of the FE model. The nominal shear strength was presented to verify the finiteness of the model:

(4) $$\tau =\frac{F_{max}}{2bl}$$

where τ denotes the nominal shear strength of DJSLs, *F_max_* denotes the ultimate load of DJSLs, and *b* and *l* denote the width and length of DJSLs, respectively.

**Figure 17:**
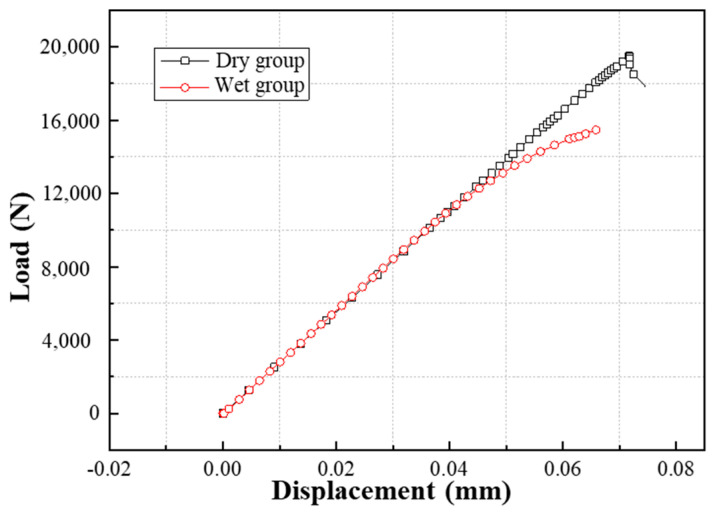
Load−displacement curves for DLSJs.

**Figure 18:**
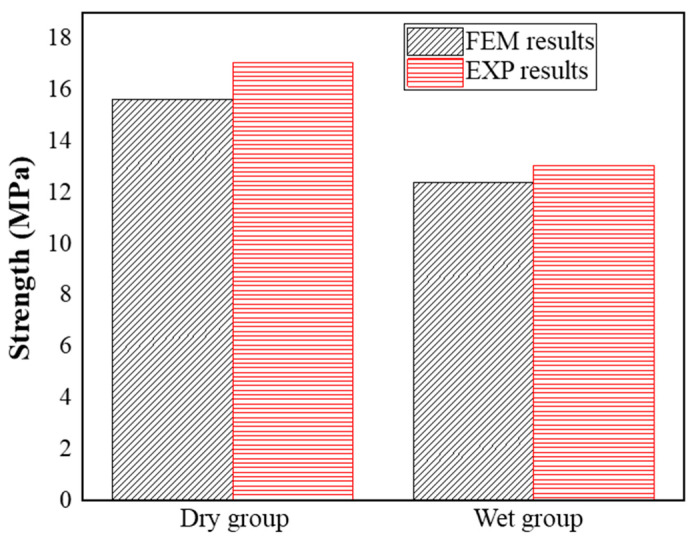
Comparison of experimental and FEM results.

**Figure 19:**
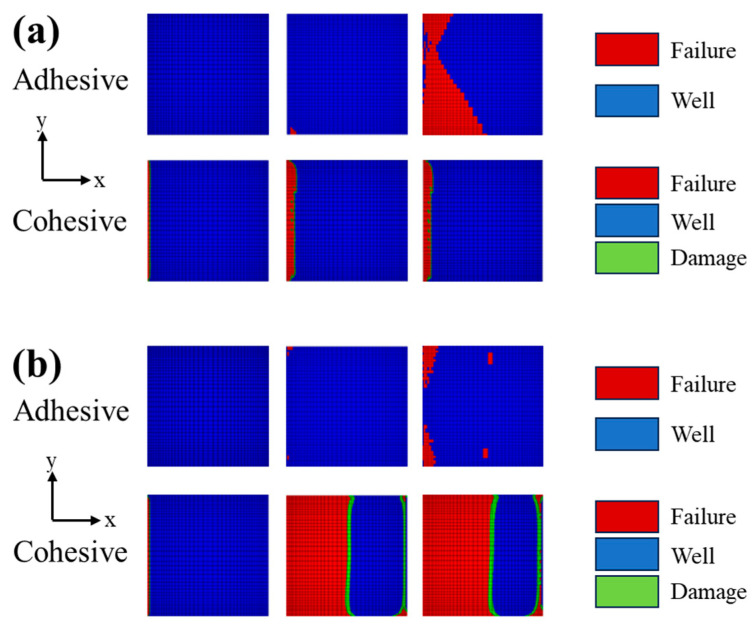
Failure modes of the DLSJs: (**a**) dry environment, (**b**) wet environment.

The tensile load−displacement curves for the DLSJs with FE analysis are depicted in Figure 17. The nominal shear strength of the DLSJ specimens is shown in Figure 18. It can be seen that the FEM was a useful tool for predicting the shear strength of the DLSJ samples under different environments, with errors of −8.5% and 5.0% for the dry and wet groups, respectively. Figure 19 shows the progress of damage evolution in the adhesive and cohesive regions. The dry group’s initial damage occurs at the left side (near the metal part) of the cohesive region (the interface between the composite and the adhesive), and when the damage expands to a certain status, the damage is transferred to the adhesive region from the cohesive region, presenting adhesive failure modes. For the wet group, the initial damage also occurs at the left side of the cohesive region, and when the damage expands to half of this region, the fracture transfers to the adhesive region.

## 5. Conclusions

An experimental study was carried out to assess the effect of wet conditions on the mechanical performance of an adhesive and interfaces in DLSJs. A series of parameters related to the adhesive and interface properties were obtained. In addition, the FE model was used to analyze the failure mode of the composite−to−metal DLSJs under tensile loading using cohesive zone modeling. The conclusions are summarized as follows:

(1) The moist environment significantly influences the tensile strength and modulus of the adhesive, decreasing by approximately 28.1% and 30.9%, respectively.
(2) In the composite/adhesive fracture energy tests, the *G_IC_* and *G_IIC_* decreased by 32.6% and 91.2%, respectively, after immersion in 95 °C water for 40 d. In the metal/adhesive fracture energy tests, the *G_IC_* and *G_IIC_* decreased by 33.5% and 67.4%, respectively, after immersion in 95 °C water for 29 d.
(3) The ultimate load of the wet group specimens decreased by almost 25.4% after immersion in 95 °C water for 50 d, while the initial stiffness (the slope of the load) of the DLSJ specimens in different environments was nearly invariable, indicating that joint strength is significantly affected by the wet environment; however, the influence on joint stiffness can be ignored.
(4) The failure modes of the DLSJs under dry and wet environments differed. The main damage was an adhesive failure in the dry group, while the interlaminar failure was the main damage in the wet group.
(5) The predicted results of the failure loads of the DLSJs under tensile loading for the dry and wet groups obtained using FEM had errors of -8.5% and 5.0%, respectively, compared with the results of the tests. The damage evolution obtained through FEM also showed good consistency with EXP.

## Figures and Tables

**Table 1:** Basic physical properties of SY−14M−III.

Properties	Value
Nominal thickness (mm)	0.13 ± 0.01
Areal density (g/m^2^)	159 ± 12

**Table 2:** Cohesive parameters used in composite/adhesive model.

	Environment	Interface Stiffness, K (N/mm^3^)	Interface Strength, σ (MPa)	Fracture Toughness, $G_{C}$ (J/m^2^)	Dispersion of *G_C_*
Mode I	Dry	112,000	22	620	3.2%
	Wet	75,000	15	440	6.1%
Mode II	Dry	80,000	68	5140	4.9%
	Wet	45,000	26	450	3.8%

**Table 3:** Cohesive parameters used in metal/adhesive model.

	Environment	Interface Stiffness, K(N/mm^3^)	Interface Strength, σ(MPa)	Fracture Toughness, $G_{C}$(J/m^2^)	Dispersion of *G_C_*
Mode I	Dry	75,000	45	2304	2.6%
	Wet	40,000	30	1510	3.5%
Mode II	Dry	75,000	95	7540	4.7%
	Wet	50,000	64	5300	5.2%

**Table 4:** Laminate parameters used in the model [6,31].

Parameters	Value
*E*_11_ (GPa)	173
*E*_22_, *E*_33_ (GPa)	8.47
*G*_12_, *G*_13_ (GPa)	4.25
*G*_23_ (GPa)	3.6
*ν*_23_, *ν*_12_, *ν*_13_	0.34

**Table 5:** Comparison of crack evolution of *G_IC_* tests between EXP and FEM results.

Types	Environments	EXP (mm)			FEM (mm)		Error (%)
		Initial Crack	Crack Evolution (Left)	Crack Evolution (Right)	Initial Crack	Crack Evolution	
Composite/adhesive	Dry	41.3	40.1	40.1	41.0	39.1	3.0
	Wet	49.3	28.1	25.3	49.0	26.4	1.1
Metal/adhesive	Dry	37.0	49.2	50.1	37.0	53.9	8.6
	Wet	37.0	54.6	55.3	37.0	62.5	13.7

## Data Availability

Data are contained within the article.
